# Knowledge-Attitude-Practice Gap Among Mothers of Under-Five Children With Iron-Deficiency Anemia and the Association of Maternal Knowledge With Hemoglobin Levels: A Cross-Sectional Study at a Rural Tertiary Care Center in Haryana

**DOI:** 10.7759/cureus.114866

**Published:** 2026-08-20

**Authors:** Ashiya Loomba, Surendra Kumar, Vishal Sharma, Sheikh Mohammad Abdur Rahman, Amanjot Singh Nokwal, Mehak Mehta, Karnika Agrawal

**Affiliations:** 1 Pediatrics, Maharaja Agrasen Medical College, Agroha, IND

**Keywords:** hemoglobin, iron deficiency anaemia, maternal knowledge, preventive practices, rural health, under 5 children

## Abstract

Background: Anemia is the third leading cause of disability globally, with iron deficiency as the most common nutritional cause. India accounts for nearly half of the global anemia burden in children under five years of age. Given that mothers play a critical role in child care, this study aimed to identify gaps in their knowledge, attitudes, and practices (KAP) regarding anemia prevention.

Method: A hospital-based cross-sectional study was conducted among 102 mothers of anemic children aged 6-59 months diagnosed with iron-deficiency anemia (IDA) and presented to a rural tertiary care center in Haryana. Participants were selected by systematic random sampling after obtaining written informed consent. Data were collected using a prevalidated, interviewer-administered, semistructured questionnaire comprising sociodemographic characteristics, knowledge, attitude, and preventive practice domains. Open-ended responses were categorized using qualitative content analysis. Knowledge scores were calculated for each participant. Pearson's correlation was used to assess the relationship between maternal knowledge scores and children's hemoglobin levels. One-way analysis of variance (ANOVA) with Tukey's honestly significant difference (HSD) post hoc test compared mean knowledge scores across anemia severity groups. Statistical significance was set at p < 0.05.

Results: Most mothers had secondary or higher education (n = 75; 73.5%) and were homemakers (n = 85; 83.3%). Over half of the participants failed to recognize the role of antenatal iron-folic acid supplementation (n = 56; 54.9%) and diet (n = 63; 61.8%) in anemia prevention. In addition, several other important knowledge gaps and misconceptions were identified. Only 41 (40.2%) recognized worm infestation as a cause of anemia, 20 (19.6%) incorrectly considered milk and milk products to be iron-rich, and only three (3%) were aware of government anemia prevention programs. Mothers generally demonstrated positive attitudes toward childhood anemia; however, preventive practices such as deworming (n = 39; 38.2%), iron supplementation (n = 25; 24.5%), and cooking in iron utensils (n = 15; 14.7%) were suboptimal. Maternal knowledge scores showed a significant positive correlation with children's hemoglobin levels (r = 0.30, p = 0.002). Mean knowledge scores differed significantly according to anemia severity (ANOVA: F(2,99) = 5.54, p = 0.005), with mothers of children with mild anemia demonstrating significantly higher knowledge scores than those of children with severe anemia (Tukey's HSD, adjusted p = 0.004).

Conclusion: Higher maternal knowledge was associated with better hemoglobin levels in their children. Despite generally favorable attitudes, substantial gaps exist between maternal knowledge and preventive practices regarding childhood IDA. This emphasizes the need for targeted educational interventions, especially in rural areas, to reduce anemia prevalence and its associated health impacts.

## Introduction

Anemia is a condition associated with reduced oxygen-carrying capacity of blood caused by the interplay of several factors, among which iron deficiency is the most common nutritional cause [1]. Anemia can also occur secondary to underlying diseases such as malaria, sickle cell disease, thalassemia, and roundworm infection. Additionally, social determinants, food practices and taboos, cultural beliefs, knowledge, and socioeconomic status also contribute to the disease burden [1,2]. According to the World Health Organization (WHO) criteria, anemia in children aged 6-59 months is classified by hemoglobin concentration as mild (10.0-10.9 g/dL), moderate (7.0-9.9 g/dL), or severe (<7.0 g/dL) [2,3].

Anemia represents an estimated 5.7% of years lived with disability, making it the third most common cause of disability [4]. Globally, anemia affects roughly 24.3% of the population, with a 39.8% prevalence in children aged 6-59 months [4-5]. Despite being the fifth-largest economy, India accounts for 54.3% of the global anemia burden in the under-five age group [5-6]. According to the Fifth National Family Health Survey (NFHS-5) prevalence of anemia in 6-59 months old children was 64.2% in the urban and 68.3% in the rural population of India. As per the state fact sheet, the prevalence of anemia in Haryana was 68.1% in urban and 71.5% in rural regions, higher than the national average [7].

Iron is a crucial micronutrient for children due to rapid growth and development, especially in the early years of life, who have high iron requirements and are thus susceptible to iron deficiency, which is linked to lethargy, poor cognitive development, and, in severe cases, altered neuronal and brain function [8,9]. In addition to being a health issue, anemia incurs an economic burden. It is noted that developing countries suffer an annual 4.5% of GDP from anemia, which is 1.18% of GDP in India [10].

Although maternal knowledge, attitudes, and practices (KAP) regarding childhood anemia have been investigated in different geographical and socioeconomic settings, limited evidence is available from rural India, particularly rural Haryana [11-18]. Furthermore, relatively few studies have examined the relationship between maternal knowledge regarding childhood anemia and the hemoglobin status of children with iron-deficiency anemia (IDA). Understanding these gaps is important because mothers play a central role in children's dietary practices, adherence to iron supplementation, and other preventive measures. Therefore, the present study was undertaken to assess the knowledge, attitudes, and preventive practices of mothers of under-five children with IDA, identify gaps between KAP, and examine the association between maternal knowledge and hemoglobin levels in children attending a rural tertiary care center in Haryana. In this study, the KAP gap was operationally defined as the observed discordance between mothers' knowledge and/or favorable attitudes and their reported adoption of appropriate preventive practices. This study was presented as a paper and secured second position at the INFUSE 2024 Conference, All India Institute of Medical Sciences, Bathinda, Punjab, India.

## Materials and methods

Study setting 

This study was conducted for a duration of three months (March to May 2024) in the Department of Pediatrics of a rural tertiary care teaching hospital in Haryana after seeking prior approval from the Institutional Scientific Research Committee and Institutional Ethical Committee (Reference number: MAMC/IEC/2023/68).

Study design

This was a cross-sectional analytic study.

Sample size

The sample size was calculated using OpenEpi (Version 3.01), an open-source, web-based epidemiologic statistics software, employing the single population proportion formula. In the absence of prior data on maternal knowledge, attitude, and preventive practices regarding IDA, a prevalence of 50% was assumed to obtain the maximum sample size. With a 95% confidence level and an absolute precision of 10%, the minimum required sample size was calculated to be 97 participants. To account for possible incomplete responses, 102 mothers were enrolled in the study.

Study population

All children aged 6-59 months who presented to the outpatient department or were admitted to the pediatric ward or pediatric intensive care unit with suspected IDA were considered eligible. Only children with a final diagnosis of IDA were enrolled in the study. Before the diagnosis was established, children underwent appropriate clinical and laboratory evaluation during outpatient or inpatient care, including hemoglobin concentration, mean corpuscular volume, mean corpuscular hemoglobin, peripheral blood smear, serum ferritin, and reticulocyte count. Investigations for other causes of anemia, including hemolytic anemia, were performed whenever indicated and were ruled out by the treating pediatrician before enrollment. Hemoglobin concentration was documented for all enrolled children and used to classify anemia severity according to the WHO criteria as mild (10.0-10.9 g/dL), moderate (7.0-9.9 g/dL), or severe (<7.0 g/dL) [3]. A serum ferritin level <12 µg/L (ng/mL) was considered indicative of depleted iron stores. The first eligible participant was selected randomly, after which every second eligible child was included until the required sample size was achieved. Children with anemia attributable to an etiology other than IDA and those whose parents refused to participate were excluded from the study.

Methods in brief

After obtaining written informed consent from either parent, eligible participants were enrolled in the study. IDA was diagnosed based on clinical findings and laboratory investigations. The study questionnaire was developed by the authors after an extensive review of the published literature and relevant guidelines on childhood IDA [11-18]. It was subsequently reviewed by departmental peers and three subject experts for content validity, and modifications were made based on their feedback to improve clarity, relevance, and comprehensiveness. The final prevalidated, semistructured questionnaire was pilot-tested among 10 mothers before the study. The reliability of the questionnaire was assessed using Cronbach's alpha, which was 0.80 for the knowledge domain, indicating good internal consistency. The questionnaire was administered in the participants' local language by a single trained interviewer to minimize inter-observer bias.

The questionnaire comprised four sections. The complete questionnaire is provided in the appendices (supplementary material). The first section collected sociodemographic and clinical information, including the child's age, hemoglobin level, maternal educational status, parental occupation, socioeconomic status, and dietary habits. The second section assessed maternal knowledge regarding IDA and consisted of 13 questions, including six closed-ended questions (scored as 1 for a correct response and 0 for an incorrect response) and seven open-ended questions (scored as 2 for a correct response, 1 for a partially correct response, and 0 for an incorrect or no response) as given in Table 9. The third section evaluated maternal attitudes toward IDA prevention using 10 statements rated on a modified five-point Likert scale ranging from 5 (strongly agree) to 1 (strongly disagree) as presented in Table 10. The fourth section assessed preventive practices related to IDA using 10 closed-ended questions (Table 11). Finally, participants were asked to identify their primary source(s) of information regarding anemia, including family or friends, healthcare professionals, mass media, Accredited Social Health Activists (ASHAs), Anganwadi workers, or other sources (Table 12).

Statistical analysis

Data were entered into MS Excel (Microsoft Corporation, Redmond, Washington, United States) and subsequently analyzed using Jamovi software (version 2.7). Participant confidentiality was maintained by anonymizing all records before analysis. Continuous variables were summarized as mean ± SD and categorical variables as frequencies and percentages. The normality of the data was assessed using the Shapiro-Wilk test and graphical methods. Homogeneity of variance was assessed using Levene's test. One-way ANOVA followed by Tukey's HSD post hoc test was used to compare mean knowledge scores among children with mild, moderate, and severe anemia. Attitude was analyzed descriptively at the item level, and no composite attitude score was calculated; reverse scoring was not performed. Pearson's correlation coefficient was used to assess the association between maternal knowledge scores and hemoglobin levels. Linearity and potential influential observations were assessed using scatterplots. Statistical significance was set at a two-sided p-value <0.05.

## Results

A total of 102 mothers of under-5 children with IDA were enrolled in the study. The baseline sociodemographic characteristics of the study participants are summarized in Table 1. Most mothers had secondary or higher education (n = 75; 73.5%), and the majority were homemakers (n = 85; 83.3%). Most participants belonged to the low socioeconomic group (n = 63; 61.8%) and followed a vegetarian diet (n = 94; 92.2%).

**Table 1:** Baseline sociodemographic characteristics of the study participants (N = 102)

Variable	Characteristic	n (%)
Age of the child	6-12 months	40 (39.2)
	1-2 years	29 (28.4)
	2-3 years	9 (8.8)
	3-4 years	7 (6.9)
	4-5 years	17 (16.7)
Education of the mother	Illiterate	17 (16.7)
	Primary education	10 (9.8)
	Secondary education	37 (36.3)
	Higher secondary education	17 (16.7)
	Graduate or above	21 (20.6)
Occupation of the mother	Homemaker	85 (83.3)
	Daily wage worker	11 (10.8)
	Self-employed	5 (4.9)
	Teacher	1 (1.0)
Socioeconomic status	Low	63 (61.8)
	Middle	39 (38.2)
	High	0 (0)
Predominant diet	Vegetarian	94 (92.2)
	Nonvegetarian	8 (7.8)

The responses to closed-ended knowledge questions are presented in Table 2. More than half of the mothers were unable to recognize the role of antenatal iron-folic acid supplementation (n = 56; 54.9%) and diet (n = 63; 61.8%) in preventing childhood anemia. However, knowledge regarding other aspects was limited. Only 41 (40.2%) identified worm infestation as a cause of anemia, and awareness of government anemia prevention programs was extremely low (n = 3; 3%). Notably, 97 (95.1%) mothers incorrectly believed that milk and curd are beneficial in treating anemia, highlighting a major misconception.

**Table 2:** Responses of mothers to closed-ended knowledge questions on childhood anemia (N = 102) *The scientifically correct response is "No"

Questions	Correct responses
	n (%)
Do antenatal iron folic acid tablets affect a child's hemoglobin?	46 (45.1)
Does diet have any role in anemia?	39 (38.2)
Do worm/parasite infections cause anemia?	41 (40.2)
Is milk/curd of any help in anemia? *	5 (4.9)
Is eating nonnutritious food (pica) associated with anemia?	51 (50.0)
Are you aware of any government programs for anemia prevention?	3 (3.0)

The responses to open-ended knowledge questions are summarized in Table 3. Content analysis revealed that only 38 (37.3%) mothers correctly described anemia as “khoon ki kami” (reduced hemoglobin levels), whereas nearly half (n = 48; 47.1%) were unable to define anemia. Pallor was the most frequently recognized symptom (n = 60; 58.8%), and poor diet was the most commonly identified cause (n = 63; 61.8%). Although almost half of the mothers identified green leafy vegetables, pulses, and fruits as iron-rich foods (n = 50; 48.0%), misconceptions regarding iron nutrition were common, with 19.6% (20) considering milk and milk products to be iron-rich. Furthermore, 97 (95.1%) mothers were unaware of dietary factors that inhibit iron absorption.

**Table 3:** Responses of mothers to open-ended knowledge questions on childhood anemia (N = 102)

Question	Mother’s response	n (%)
What is anemia according to you?	Khoon Ki Kami (blood deficiency)	38 (37.2)
	An illness	5 (4.9)
	Kamzori (weakness)	11 (10.8)
	Don’t know	48 (47.1)
How to identify anemia?	Pale discoloration	60 (58.8)
	Weakness	19 (18.6)
	Poor appetite	3 (2.9)
	Diarrhea and fever	3 (2.9)
	Don’t know	17 (16.7)
Possible causes of anemia?	Poor appetite	63 (61.8)
	Diarrhea and fever	8 (7.8)
	Don’t know	31 (30.4)
What are the effects of anemia in child?	Lethargy	56 (54.9)
	Poor growth	3 (2.9)
	Child will get sick	14 (13.7)
	Diarrhea and fever	8 (7.8)
	Vertigo	4 (3.9)
	Poor immunity	3 (2.9)
	Don’t know	14 (13.7)
Name some iron-rich food	Fruits, pulses, and green vegetables	49 (48.0)
	Milk and milk products	20 (19.6)
	Jaggery Chana	4 (3.9)
	Don’t know	29 (28.4)
Can food inhibit iron absorption? Name if any	Milk	2 (2.0)
	Junk food	3 (2.9)
	Don’t know	97 (95.1)
What are the complications of anemia?	Poor growth	48 (47.1)
	Illness	19 (18.6)
	Poor immunity	3 (02.9)
	Death	2 (2.0)
	Don’t know	30 (29.4)

One-way analysis of variance (ANOVA) demonstrated a significant difference in mean maternal knowledge scores according to the severity of anemia among under-five children (Table 4). Tukey's honestly significant difference (HSD) post hoc analysis showed that the mean knowledge score was significantly higher among mothers of children with mild anemia compared with those of children with severe anemia. However, the differences between the mild and moderate anemia groups and between the moderate and severe anemia groups were not statistically significant (Table 5).

**Table 4:** Comparison of the mean maternal knowledge scores according to severity of anemia among under-five children SD: standard deviation; ANOVA: analysis of variance p-values were obtained using one-way ANOVA. Statistical significance was set at p < 0.05. F denotes the ANOVA Fisher's F statistic. Degree of freedom (df1 = 2; df2 = 99)

Severity of anemia	n (%)	Mean knowledge score ± SD	One-way ANOVA*
Mild	20 (19.6)	11.35 ± 3.58	F (2,99) = 5.54; p-value = 0.005
Moderate	56 (54.9)	9.52 ± 3.62	
Severe	26 (25.5)	7.92 ± 3.60	

**Table 5:** Tukey's HSD post hoc comparison of maternal knowledge scores according to the severity of anemia HSD: honestly significant difference; ANOVA:; analysis of variance; CI: confidence interval Pairwise comparisons were performed using Tukey's HSD post hoc test following one-way ANOVA. Mean differences are presented with 95% CI and adjusted p-values. Statistical significance was defined as p < 0.05

Pairwise comparison	Mean difference	95% CI	Adjusted p-value
Mild vs moderate	1.83	-0.32 to 3.98	0.110
Mild vs severe	3.43	0.97 to 5.88	0.004
Moderate vs severe	1.59	-0.36 to 3.55	0.133

**Figure 1:**
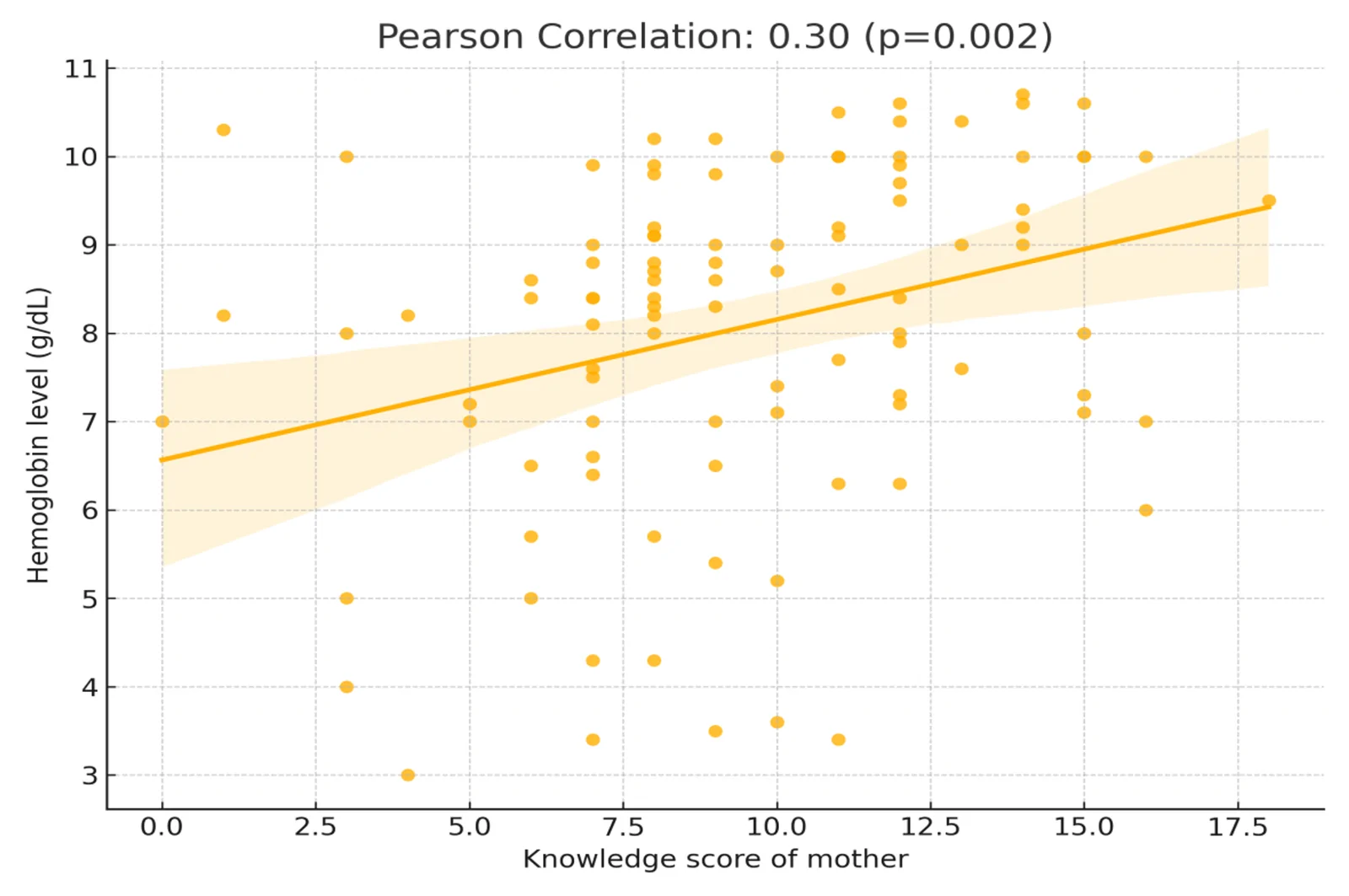
Scatter plot showing the correlation between maternal knowledge score and hemoglobin level among under-five children The solid line represents the fitted linear regression line with the 95% confidence interval. Pearson's correlation coefficient: r = 0.30; p = 0.002

Pearson's correlation analysis demonstrated a statistically significant positive correlation between maternal knowledge scores and hemoglobin levels among under-five children (r = 0.304; df = 100; p = 0.002), indicating that higher maternal knowledge was associated with higher hemoglobin levels (Figure 1).

The attitude of mothers toward childhood anemia was generally favorable (Table 6). Most mothers perceived anemia as a serious health problem (n = 90; 88.2%), acknowledged the importance of dietary iron (n = 92; 90.2%), and supported regular screening of children for anemia (n = 93; 91.2%). In addition, 85 (83.3%) mothers agreed that anemia adversely affects children's growth and development, while 60 (59.8%) mothers believed that anemia is preventable. Nevertheless, misconceptions regarding the association between a predominantly milk-based diet and anemia remained common, with only 20 (19.6%) mothers recognizing this relationship.

**Table 6:** Responses of mothers to questions assessing attitudes towards childhood anemia (N = 102)

Questions	Agree	Neutral	Disagree
	n (%)	n (%)	n (%)
Your child is having anemia	64 (62.7)	9 (8.8)	29 (28.4)
Anemia is a problem of concern at community/national level	77 (75.5)	14 (13.7)	11 (10.8)
Anemia can be prevented	61 (59.8)	10 (9.8)	31 (30.4)
Anemia can cause serious problem in child	90 (88.2)	8 (7.8)	4 (3.92)
Anemia can be associated with other diseases	52 (51.0)	30 (29.4)	19 (18.6)
Milk predominant diet can cause anemia	20 (19.6)	5 (4.9)	77 (75.5)
Iron is an important part of diet	92 (90.2)	10 (9.8)	0 (0)
Anemia is easy to treat	60 (58.8)	12 (11.8)	30 (29.4)
Children should have regular check-up for anemia	93 (91.2)	7 (6.9)	2 (2.0)
Anemia hampers growth and development of child	85 (83.3)	9 (8.8)	8 (7.8)

The preventive practices adopted by mothers are summarized in Table 7. Overall, appropriate dietary practices were common, with most mothers reporting frequent consumption of green leafy vegetables (n = 84; 82.4%), locally available fruits (n = 87; 85.3%), and citrus fruits or other vitamin C-rich foods (n = 75; 73.5%). The majority also reported limiting junk food consumption (n = 88; 86.3%). However, several gaps in preventive practices were also identified. Only 39 (38.2%) mothers practiced regular deworming, 25 (24.5%) administered iron supplements to their children, and 15 (14.7%) cooked food in iron utensils. In addition, 64 (62.7%) mothers reported serving tea or coffee with or immediately after meals, a practice that may impair iron absorption.

**Table 7:** Mothers' preventive practices related to childhood anemia (N = 102)

Questions	Inappropriate practice	Appropriate practice
	n (%)	n (%)
Do you serve tea/coffee with/after meals?	64 (62.7%)	38 (37.3%)
Do you cook food in iron vessel?	87 (85.3%)	15 (14.7%)
Do you practice deworming?	63 (61.8%)	39 (38.2%)
Do you allow junk food frequently?	14 (13.7%)	88 (86.3%)
Do you use iron supplements for child?	77 (75.5%)	25 (24.5%)
Do you serve citrus fruits/vitamin C?	27 (26.5%)	75 (73.5%)
Do you consume meat/poultry?	94 (92.2%)	8 (7.8%)
Do you consume green leafy vegetables?	18 (17.7%)	84 (82.4%)
Do you use jaggery in diet?	42 (41.2%)	60 (58.8%)
Do you consume any locally available fruits?	15 (14.7%)	87 (85.3%)

The reported sources of information on childhood anemia are presented in Table 8. Doctors were the most common source of information (n = 76; 74.5%), followed by ASHA/Anganwadi workers (n = 26; 25.5%) and friends or family members (n = 20; 19.6%). Mass media (n = 8; 7.8%) and other sources (n = 4; 3.9%) contributed minimally to mothers' awareness of childhood anemia.

**Table 8:** Sources of information on childhood anemia among mothers (N = 102) ASHA: Accredited Social Health Activist *Multiple responses were allowed; therefore, percentages do not total 100%

Sources of information	n (%)*
Doctor	76 (74.5)
ASHA/Anganwadi workers	26 (25.5)
Friends/family	20 (19.6)
Mass media	8 (7.8)
Other sources	4 (3.9)

## Discussion

The present study explored maternal knowledge, attitudes, and preventive practices regarding childhood IDA and their relationship with the hemoglobin status of their anemic under-five children. The findings revealed substantial deficiencies in maternal knowledge and preventive practices despite generally favorable attitudes towards childhood anemia. Furthermore, maternal knowledge demonstrated a significant positive association with children's hemoglobin levels, suggesting that improving maternal awareness may contribute to better anemia-related outcomes.

In the present study, 38 (37.3%) mothers correctly defined anemia as reduced hemoglobin levels ("khoon ki kami"), whereas 48 (47.1%) had never heard of anemia or were unable to define it. These findings are consistent with the study conducted by Samararathna et al. in Sri Lanka, where only 33% of mothers had an accurate understanding of anemia and 27.4% were unaware of the condition [11]. Similarly, Souganidis et al. from Indonesia reported that only 37% of mothers possessed adequate knowledge regarding anemia [12]. Pallor was identified as the most common symptom of anemia by 60 (58.8%) mothers in our study, which is comparable to the findings of Anokye et al., where 47% and 44% of mothers recognized pale conjunctiva and pallor, respectively, as important clinical indicators of anemia [13], Likewise, Samararathna et al. reported that fatigue was the most commonly recognized symptom, while 43.9% of mothers were aware that anemia could impair child development [11]. In the present study, nearly half of the mothers (n = 48; 47.1%) recognized poor growth as a consequence of untreated anemia, reflecting a moderate awareness of its adverse effects on child health.

Poor dietary intake was identified as the principal cause of anemia by 63 (61.8%) mothers, consistent with observations reported by Awuah et al. [14]. However, important misconceptions regarding dietary practices persisted. Although most mothers acknowledged the role of diet in anemia prevention, 97 (95.1%) were unaware of foods that inhibit iron absorption, with only 2 (2.0%) correctly identifying milk as a dietary inhibitor, possibly explaining why 64 (62.7%) mothers continued to serve coffee/tea along with meals. In contrast, Samararathna et al. reported substantially better awareness, with 64.5% and 8.7% of mothers identifying tea and milk, respectively, as inhibitors of iron absorption [11]. These findings suggest that while general nutritional awareness exists, mothers often lack specific knowledge regarding appropriate dietary practices that optimize iron absorption, which may partly explain the continued burden of childhood anemia despite apparently favorable attitudes.

An important finding of the present study was the statistically significant positive correlation between maternal knowledge scores and children's hemoglobin levels (Pearson's r = 0.30, p = 0.002). Furthermore, mean maternal knowledge scores differed significantly across anemia severity groups (one-way ANOVA: F(2,99) = 5.54, p = 0.005), with mothers of children having mild anemia demonstrating significantly higher knowledge scores than those of children with severe anemia on Tukey's HSD post hoc analysis. Similar observations were reported by Bilenko et al., who demonstrated a positive association between maternal knowledge and children's hemoglobin status [15]. These findings emphasize that maternal knowledge may play an important role in influencing child nutritional practices and anemia prevention, although the observed correlation was modest, indicating that additional socioeconomic, dietary, environmental, and healthcare-related factors also contribute to childhood anemia.

Although maternal attitudes toward childhood anemia were generally positive, this favorable attitude was not consistently reflected in preventive practices. Approximately 60% of mothers believed that anemia is preventable, 88.2% (n = 90) recognized its serious health consequences, and over 90% acknowledged the importance of dietary iron and regular health check-ups. Nevertheless, only 20 (19.6%) mothers recognized that a predominantly milk-based diet could contribute to anemia. Moreover, despite 75 (73.5%) mothers having attained secondary education or higher, substantial gaps in preventive practices persisted. Similar findings were reported by Al-Suhiemat et al., who observed a high prevalence of childhood anemia despite maternal education, largely attributable to inappropriate feeding practices and inadequate consumption of iron absorption enhancers [16]. These findings suggest that formal education alone may not translate into appropriate health behaviors unless accompanied by targeted nutrition education and behavior change communication.

The present study also identified several deficiencies in maternal preventive practices. Nearly two-thirds of mothers reported serving tea or coffee with or immediately after meals, a practice known to reduce dietary iron absorption, while only 39 (38.2%) practiced regular deworming and only 25 (24.5%) mothers administered iron supplements to their children. Conversely, encouraging dietary practices such as regular consumption of green leafy vegetables, vitamin C-rich foods, and locally available fruits were reported by the majority of mothers. Similar observations were made by Pasricha et al., who demonstrated inadequate iron supplementation among children in rural India, with only 41.5% receiving iron supplements and very few obtaining them through the public health system [17]. These findings indicate that improving the availability, accessibility, and utilization of iron supplementation programs remains an important public health priority.

Healthcare providers emerged as the principal source of maternal information regarding anemia in the present study, with doctors followed by ASHA and Anganwadi workers being the most frequently cited sources. Similar findings have been reported by Guedenon et al., where healthcare workers were the primary source of information and mass media contributed minimally to anemia awareness [18]. In line with the findings reported by Gupta et al., strengthening counseling provided by frontline healthcare workers and integrating consistent anemia-related health education into community-based programs may therefore represent an effective strategy for improving maternal knowledge and translating positive attitudes into appropriate preventive practices [19]. Similarly, a recent population-based analysis from Nepal identified maternal anemia as a significant predictor of childhood anemia, further emphasizing the influence of maternal factors on children's hemoglobin status [20].

The principal strength of the present study lies in its comprehensive assessment of maternal knowledge, attitudes, and preventive practices using a prevalidated questionnaire containing both closed- and open-ended questions, together with the evaluation of their association with objectively measured hemoglobin levels among children. In addition, the use of correlation analysis and ANOVA provided quantitative evidence linking maternal knowledge with childhood anemia severity.

The study has certain limitations. Being a single-center, hospital-based cross-sectional study, the findings may not be generalizable to the wider community, and causal inferences cannot be established. The hospital-based recruitment of children with IDA may have introduced selection bias. Maternal knowledge and preventive practices were self-reported and may therefore have been affected by recall and social-desirability bias. Although open-ended responses were scored using predefined criteria, some subjectivity in categorizing such responses cannot be completely excluded. Furthermore, the reported association between maternal knowledge and children's hemoglobin levels was not adjusted for potential confounding factors such as socioeconomic status, dietary diversity, maternal characteristics, or other environmental and healthcare-related determinants. Paternal knowledge was also not assessed. Future multicenter community-based studies with larger sample sizes and inclusion of both parents are warranted. Future multicenter community-based studies with larger sample sizes and inclusion of both parents are warranted to better understand the determinants of childhood anemia and to evaluate interventions aimed at improving maternal knowledge and preventive practices.

## Conclusions

This study demonstrated that although mothers of under-five children with IDA generally exhibited favorable attitudes toward anemia, significant gaps persisted in their knowledge and preventive practices. Higher maternal knowledge was significantly associated with better hemoglobin levels in children, highlighting the potential influence of maternal awareness on childhood anemia outcomes. Misconceptions regarding dietary practices, poor awareness of government anemia prevention programs, inadequate deworming, and low utilization of iron supplementation indicate the need for targeted behavior change interventions. Strengthening maternal nutrition education through healthcare providers and frontline health workers, along with improving the implementation of existing anemia control programs, may enhance preventive practices and contribute to reducing the burden of childhood IDA.

## Appendices

Study proforma and questionnaire

Section 1: Sociodemographic Details

Age of child:

Hemoglobin level of the child:

Education of Mother: Illiterate/Primary/Secondary/Higher secondary/Graduate or above

Occupation of Mother: Low/Middle/High

Socioeconomic status:

Diet: Vegetarian/nonvegetarian

Section 2: Knowledge of Mothers

**Table 9:** Knowledge questionnaire and scoring criteria for mothers of anemic children aged 6-59 months Closed-ended questions were assigned 1 mark each, and open-ended questions were assigned 2 marks each (total score = 20)

Question	Response		Maximum Marks
Does antenatal iron-folic acid affect child Hb?	Yes	No	1
Does diet have any role in anemia?	Yes	No	1
Do worm/parasitic infestations cause anemia?	Yes	No	1
Is milk/curd/lassi of any help in anemia?	Yes	No	1
Is eating non-nutritious food associated with anemia?	Yes	No	1
Aware of any govt program for anemia?	Yes	No	1
What is anemia according to you			2
How to identify anemia? (symptoms of anemia)			2
Possible causes of anemia			2
Importance of normal Hb/ effects of anemia on child			2
What food are rich in iron?			2
Can food inhibit iron absorption? Name if any			2
Complication of anemia?			2

Section 3: Attitude of Mothers on Likert Scale

**Table 10:** Attitude questionnaire for mothers of anemic children aged 6-59 months

Questions	Strongly agree	Agree	Neither agree nor disagree	disagree	Strongly disagree
Your child is having anemia	5	4	3	2	1
Anemia is a problem of concern at community/national level	5	4	3	2	1
Anemia can cause serious problem in child	5	4	3	2	1
Anemia can be prevented	5	4	3	2	1
Anemia is associated with other diseases	5	4	3	2	1
Milk predominant diet can cause anemia	5	4	3	2	1
Iron is important part of diet	5	4	3	2	1
It is difficult to treat anemia	5	4	3	2	1
Children should have a regular check-up for anemia	5	4	3	2	1
Anemia may hamper normal growth and development of child	5	4	3	2	1

Section 4: Preventive Practices by Mothers 

**Table 11:** Preventive practices questionnaire for mothers of anemic children aged 6-59 months

Questions	Response	
Do you serve tea/coffee with/after meal?	Yes	No
Do you cook food in iron vessel?	Yes	No
Do you practice deworming?	Yes	No
Do you allow junk food frequently?	Yes	No
Do you use iron supplement for child?	Yes	No
Do you serve citrus fruits?	Yes	No
Do you consume meat/poultry/fish?	Yes	No
Do you consume green leafy vegetables?	Yes	No
Do you use jaggery?	Yes	No
Do you consume any locally available fruits?	Yes	No

Section 5: Source of Information for Mothers About Anemia Prevention

**Table 12:** Sources of information on anemia prevention for mothers of anemic children aged 6-59 months ASHA: Accredited Social Health Activist Percentages may exceed 100% because multiple responses were permitted

Source	Response (participants can select more than one response​​​​)
Doctor	
ASHA/Anganwadi workers	
Mass media	
Friends/family	
Other sources	
